# Medical and Physiological Problems

**Published:** 1846-01

**Authors:** 


### Medical and Physiological Problems, being chiefly Researches for correct Principles of Treatment in Disputed Points of Medical Practice. By *William Griffin*, M.D., and *Daniel Griffin*, M.D. Octavo, pp. 258. London, Sherwood, 1845.

The Essays which constitute this volume originally appeared, we believe, in the pages of our Dublin cotemporary. Although the reproduction of such detached papers is usually inexpedient, such is not the case with the present ones, since they abound in useful practical remarks, and are illustrated by numerous interesting cases, detailed with a graphic power which seems almost peculiar to the authors' countrymen. With the exception of two or three, they are from the pen of Dr. William Griffin, Physician to the Limerick Infirmary, who thus states the view he had in their production.

"There are two *modes* of treating all diseases, a right and a wrong one; and it is popularly believed that the science of medicine has long since determined between them; that in every dangerous case, or, at all events, in those of ordinary or frequent occurrence, the practitioner has only to refer to received principles or authorities on the subject, and that if he commits an error in his selection of remedies, it is entirely attributable to his want of information or ability. It would, no doubt, astonish the public very much, and do little credit to medical art, if they were told, that there is scarcely a complaint to which humanity is liable, the treatment of which is, in all points, absolutely agreed upon by the profession; and that with reference to the most ordinary of dangerous cases, and those supposed to be best understood, remedies of a different and even opposite nature have been advocated by men of the highest celebrity. * * *

"It occurred to me some years since, that it would be a most useful, practical, and interesting study to collect cases with reference to all the most important of the disputed points in practice, and afterwards as a sufficient degree of personal experience happened to be attained on any one of them, to review the opinions of all the best authors on the subject, and endeavour to solve that most difficult question—'What is the correct principle of treatment?'"—*Preface.*

We may now advert to some of the results of this system of inquiry.

1. *What Principles should be kept in view by the Physician in the Treatment of Enteritis?* After adverting to the discrepancy which prevails in the advice given by the highest authorities as to the employment of bleeding (at least in the advanced stages), and the administration of purgatives in peritoneal enteritis, Dr. Griffin insists upon the importance of proportioning the *venæsection* to the strength of the patient—this being estimated by his state of health prior to the attack rather than by his condition in the early stage of the disease. Unless this rule be observed, we risk confounding indirect debility produced by the depressing influence exerted by the disease on the powers of life in a robust subject, for the true debility which is present when causes of exhaustion, as hæmorrhage, &c., have preceded the attack of inflammation. In an interesting case, related at great length, the patient, of a hysteric yet strong habit, at first fainted after a few ounces of blood were taken, but as the inflammation progressed, bore much fuller depletion well; so that the propriety of placing persons in the erect posture for the readier induction of syncope, as recommended by Elliotson and others, is questionable in some cases; since, however serviceable syncope, occurring after a small loss of blood, may prove in colic, it is only of temporary avail in inflammatory action. "There must be an absolute abstraction of blood, capable of directly depressing the whole system, to produce any permanent influence on the disease." So, too, Dr. M. Hall's proposal of taking the power which the system exhibits of resisting syncope in bleeding in this posture as a measure of the extent of inflammatory action present, is seldom applicable at the *commencement* of the disease, when such a test is most wanted. It is only as the disease advances that the inflammatory characters predominate over those of the individual habit. But, if bleeding has not been freely performed at the commencement of the disease, caution is required in its institution afterwards; for, although the author is scarcely inclined to agree with those who think that gangrene is thus more readily induced, yet he is not disposed to close his ears to the opinions of so many able practitioners who have denounced the employment of bleeding at a late period, when the disease seems to have acquired a power of vitality not to be subdued in the same manner by depletion as on the first day of the attack. Early and free bleeding having failed, or been neglected, venesection henceforward becomes only an auxiliary, not a principal means.

Dr. Griffin joins those who object to the administration of *Purgatives* in enteritis, and believes that "*people do not recover because they are purged, but they are purged because they recover.*" Inflammatory action being subdued, a lax state of bowels follows, which it might have been not only difficult to have procured before this by purgatives, but dangerous to attempt doing so, in consequence of the easy propagation of irritation from the mucous to the serous surface of the bowels—a danger which precludes our having recourse to the derivative purging so useful in cephalic and thoracic inflammations.

Of the use of *Opium* in peritoneal enteritis, Dr. Griffin entertains the highest opinion, and relates several cases in which very large and frequent doses were given with the happiest results. In one case, first two grains and then one grain doses were given every two hours, until 32 grains had been taken, two or three grain doses being resorted to on the occurrence of a relapse. In the case of a girl, 10 years of age, whose condition had been previously much aggravated by the use of purgatives, and who appeared to be sinking, 20 drops of laudanum were given, and in half an hour a grain of opium. Sound sleep ensued, and the patient who had seemed almost moribund was saved, the opiate being continued for some time at longer intervals. To a boy, æt. 5, in whom peritonitis occurred during the last stage of typhus fever, probably from perforation, grain doses were given with a successful result. Dr. Griffin does not propose opium as a substitute for general or local bleeding where these can be borne, but as a most useful remedy where this is not the case; or where the disease continues in spite of their institution. The state of the bladder should be carefully watched, as retention of urine is not of unlikely occurrence during the use of full opiates. We may extract some of the concluding remarks.

"It must not be imagined, from all I have stated, that I believe *purgatives*

to be at all times unadvisable in enteritis. There are perhaps occasions in which they are of use even in the early stage, but it is difficult to offer indications by which we shall recognize these occasions in practice. In the advance of the disease, when its force is considerably broken, and the bowels may be supposed capable of acting without increasing or renewing the inflammation, there must be an obvious advantage in getting rid of the contents of the bowels, and this may perhaps be then generally effected with safety by means of mild purgatives, combined with henbane. If, however, there was no injurious distension present, and the inflammation was progressively declining, my disposition would be, to await a more perfect amendment before I would give even these. * * * Without pretending to have satisfactorily solved the problem which I have yet ventured to discuss at such length, I shall merely recapitulate a few of the principal facts, as far as they appear to be such, and leave the inferences to the reader. General experience testifies, that the strongest purgatives will not operate in the early stages of inflamed bowels, unless large depletion by the lancet has been premised, that is, unless the violence of the inflammation has been in some measure subdued; while, on the other hand, as soon as this has been accomplished they commonly occur spontaneously, or with the assistance of the mildest purgatives. Notwithstanding the free operation of purgatives at an early stage of enteritis, the inflammation may proceed to a fatal termination, unless arrested by other remedies. A purgative has been known to occasion inflammation of the bowels, and when inflammation has been subdued by other remedies, it has brought on a recurrence of it. Inflammation of the bowels may be perfectly subdued without any evacuation at all. The bowels may even sometimes continue in a confined state for three or four days after the inflammation has subsided, without occasioning injurious distension." P. 34.

2. *Nervous Affections of the Abdomen &c. distinguished from Inflammatory.*—Dr. Griffin observes that the importance attached by Abercrombie to the existence of tenderness of the abdomen on pressure as a sign of inflammation, is in accordance with the general opinion of the profession; but that there are cases in which this exists independently of any inflammatory action; and which are to be especially distinguished, as he pointed out several years since, by the simultaneous existence of *tenderness of a corresponding portion of the spinal column.* "Wherever spinal tenderness exists, we must at all events set down pain and tenderness as wholly valueless; inasmuch as, whether there be inflammation or not, these are not peculiarly the results of it, but may also arise from the tender state of the cord. I might perhaps go much farther and assume, that where spinal tenderness exists, there also exists a state of the system scarcely compatible with acute inflammation." Several cases are given in which this symptom led to the adoption of an appropriate treatment, in lieu of the depletion seemingly indicated by the tenderness of the abdomen.

"Perhaps it may be said, that an experienced eye would have detected some anomalous symptoms in all these, which would have led to doubts of their inflammatory nature. I am not disposed to deny that much may be inferred in such attacks from the suddenness with which violent symptoms supervene, the absence of deep distress and anxiety of countenance, and above all from a freedom in the movements of the lower extremities, unusual in acute abdominal inflammations. There was indeed at least one of these discrepancies observable in a greater or lesser degree in each of the cases detailed; but how are young practitioners to form a diagnosis on such grounds. People suffer similar degrees and kinds of pain with very dissimilar degrees of fortitude, and at all events any reasoning on such signs must be founded on comparisons, which, to be worth anything, would imply an experience no young practitioner can be supposed to possess. * * * * * * * * The observations offered respecting the diagnosis of cases resembling acute inflammations, apply with almost equal truth to those resembling chronic diseases. I mean those pains affecting the chest, attended by cough and perhaps oppression, leading to apprehensions of phthisis; affecting the side below the false ribs, and suggesting affections of the liver or bowels; or affecting the pubic region and simulating disease of the womb or ovaries. The deducing inferences from acute or chronic pain apparently affecting any viscus, without examining the state of the spinal cord, seems to me little less absurd than omitting to examine the state of the hip-joint in those painful affections of the knee, the nature of which is not immediately obvious." P. 48.

The importance of this subject to young practitioners can hardly be overstated. Accustomed during their studies to hear clear, well-defined, descriptions of disease, and to witness in the hospitals well-marked examples of inflammatory affections; they are often, at the commencement of their career, ill-fitted to cope with the hysterical, mimotic and hybrid forms of disease that await them in private practice; and too often signalize it by an injudicious wasting of blood in subjects who can ill bear the loss. Although, too, the diagnostic mark, first proposed we believe by Dr. Griffin, and since frequently recommended by others, has its value in many cases; many others will still be found in which it is wanting, and yet in which the symptoms are but simulatory of inflammatory action. Some additional observations conclude a second paper upon the same subject.

"In determining the diagnosis of abdominal inflammation, where both pain and tenderness on pressure exist, we should always endeavour to ascertain—1. Whether there be any pain or tenderness on pressure in the corresponding portion of the spinal column: because, if there be, although it may not absolutely decide whether inflammation be present or not, it is quite sufficient to account for both the pain and tenderness, without assuming the existence of any inflammation. 2. Whether, if there be no spinal tenderness or pain, the soreness of the abdomen be superficial or deep-seated, which may be ascertained with tolerable certainty in all cases, by an examination directed to that end. And whether, if both superficial and deep-seated, as it usually is in peritoneal inflammation, gentle, steady, pressure with the flat of the hand can be easier borne, than with the points of the fingers. In pain and soreness from affection of the spinal nerves it commonly can be so borne, while in peritonitis every kind of pressure, and even the weight of the bed-clothes, is very distressing. (And yet one of the best means of distinguishing hysterical tenderness will often be found to be the observation of how the *slightest* degree of pressure gives rise to the expression of intense suffering, although the countenance does not always corroborate this. The tenderness in these cases is quite cutaneous.—*Rev.*) 3. Whether the boundaries of the pain or soreness extend beyond what the suspected inflammation could produce. Thus, if inflammation of the liver be suspected, and we find the soreness extending to the ileum or groin, or to the opposite side of the abdomen, it is obvious the soreness cannot be attributable to mere disease of that organ. Again, if the whole abdomen be tender to the touch in a case otherwise closely resembling peritonitis, and we find the tenderness is not confined to the abdomen, but extends over the hips and lower extremities, it is obvious, we can attach no importance to the abdominal soreness as a sign of inflammation. (This is a most valuable sign from which we have often derived the greatest assistance. *Rev.*) Finally, It should be recollected that constipation may depend on mere loss of power in the intestinal nerves, as well as on spasm, obstruction, or inflammation, since the treatment in each case must necessarily be modified, or directed by the supposed cause of this symptom." P. 59.

3. The Fourth Problem entertains the question of the reality of affection of the spinal cord or its membranes in disorders attributed to spinal irritation. Dr. Griffin quotes from a former work a tabular view of 148 cases, in which, according as the tenderness was cervical, dorsal, or lumbar, the symptoms were referrible to portions of the body supplied with spinal nerves from these respective regions. But the proof of the truth of the doctrine does not rest upon the existence of any such tenderness. In spinal irritation, according to the part implicated, we have in fact simulation of all the diseases of the economy; and a perusal of Abercrombie's work exhibits the extent. This is true also in organic disease of this part. This doctrine, too, affords a rationale of many symptoms otherwise inexplicable; and the author strongly states the great benefit which has accrued to him in the diagnosis and treatment of various affections, by observing whether tenderness is produced by pressure over the origins of the spinal nerves, and refers to cases where, by neglecting the indications thence derivable, experienced practitioners have fallen into grievous errors.

4. *Bloodletting in Diseases of the Brain.*—This is a very good paper from the pen of Dr. Daniel Griffin. The variety of opinion held by celebrated authors as to the propriety of free depletion in apoplexy is very great, although certainly the disposition among our best practitioners of the present day is to discountenance that free and indiscriminate use of the lancet, once believed, and still believed by the public at large, indispensable in such cases. Notwithstanding this opinion prevails, its practical enforcement is difficult, owing to the circumstance of cases occurring which vary greatly as to the quantity of blood they require, or will bear, to be abstracted, and yet do not present characteristic symptoms of such differences.

"In directing attention to the utter impossibility of inferring from any group of symptoms, the precise seat or the degree of any cerebral disease, I am sensible that I only point to a difficulty without showing the means of escaping from it: yet, there is one conclusion of immense practical importance that I think will be found correct, namely, that in considering the expediency of bleeding the *actual degree of organic disease that exists* is of infinitely more importance than its seat or nature, in other words, that bleeding is badly borne in all cases of extensive organic disease of the brain, *wherever the disease may be situated or whatever be its nature.* I do not deny that situation may be of much consequence in questioning the value of this remedy—our knowledge of the functions of some parts of the brain would tend to show that it is so, but we are not yet in possession of facts sufficient to prove its relative importance, and even if we should be able yet to ascertain this, the tendency of such a discovery would only be to narrow the limits of those cases in which bleeding is useful. The great mistake made about bleeding in diseases of the brain is looking to the fit as the test of its utility, instead of looking to the whole course of the disease and the event, and comparing cases in which it is used with those in which it is omitted, in all their circumstances, more particularly the last." P. 79.

Dr. D. Griffin adds, that the cases calling for large bleeding, are those of recent origin, in which little disorganization has yet been sustained by the brain; and he instances the great extent to which bleeding in affection of the brain from poisoning by opium may be advantageously carried. Our object should then be to examine what evidence there is of the existence of organic disease, and to act cautiously in proportion as this is strong. The following is the summary of reasons against the general employment of large bleedings.

"1. They are improper, because symptoms are no certain test of the amount of disease, and we may produce a degree of debility (as in a case given) that cannot be contended with. 2. They are improper in all cases in which extensive disease of the brain is known or suspected to exist; as in such cases, besides the immediate danger, they produce a degree of debility that would interfere with the process of reparation so far as that is possible. 3. They are improper in all cases of disease of the brain attended with severe and protracted pain, as such cases usually die, not from any mechanical effect of an existing inflammation, but from the exhaustion produced by the pain that accompanied it.

"As a general rule I would, therefore, even in circumstances in which the loss of a large quantity of blood was supposed to be necessary, prefer taking it away in moderate quantities at certain intervals, watching the progress of the case, and being guided entirely by it." P. 87.

5. *On what Morbid State does the occurrence of Coma and Sudden Death in Jaundice depend?*—The remarks in this paper are based upon four cases occurring in the same family within a short period of each other, in two of which the coma was followed by death, and in none of which were there any remarkable symptoms indicative of the approaching danger. Free purging proved of great service in the two cases that recovered. This complication has been very little dwelt upon by authors, and not even noticed in several even modern treatises on Jaundice. The mutual sympathies so markedly existing between the brain and the liver have led to doubts as to which organ is here primarily affected. Dr. Griffin is not disposed to agree with those who suppose these effects result from retention of bile in the circulation and its sedative influence on the brain, seeing that they bear no proportion to the frequency and extent of this occurrence.

"There are very many interesting facts which would tend to shew, that the brain is, in some instances at least, the organ primarily in fault in jaundice. Besides the well known occurrence of abscesses, and other diseased states of the liver from injuries of the head, sudden yellowness of the whole person has not unfrequently followed intense mental emotion, and has often been observed in fevers and other diseases, in which the brain and nervous system have been much affected. Some of the cases published by Dr. Marsh, to which I have already adverted, seem to have depended on an affection of the liver, and of the mucous coat of the intestines, originating in cerebral disease. If, in such instances, we could suppose the affection of the head to be so obscure as altogether to escape the attention of the practitioner, previous to the occurrence of jaundice, there would be little or no indication of an unusually dangerous form of the disease. He would almost necessarily attribute the headache, languor, and sickness to the retention of bile in the circulation, and the supervention of coma and apoplexy would seem sudden and unaccountable; when, if he could have suspected the source of the disease, it would have been anticipated as a very probable termination.

"We have not, unfortunately, a sufficient number of reports of post-mortem examinations in those cases, to form any decided opinion on the subject. If, with such imperfect materials, even a conjecture might be hazarded, I should, on the whole, be disposed to say, that the cerebral affection is rarely the primary disease, but is superinduced, we know not how, by the suppression of a most important excretion, as it sometimes is in the suppression of the catamenia, and almost always of the urine." P. 96.

6. *Is Laryngismus Stridulus, or the Crowing Disease, a Spasmodic or Paralytic Affection?*—Dr. Griffin contributes some additional cases of this singular disease. In two of these, infants of a few days old, the crowing was merely incidental, coming on during the existence of an apoplectic condition, an assemblage of symptoms to which the author gives the name of *crowing apoplexy of infants.* Both patients died. In one case, a general ramolissement of the brain was found, while, in the other, this organ was perfectly healthy. Prior to the attack the children seemed healthy, with the exception of some derangement of the bowels. The four other cases were examples of *laryngismus stridulus* so well described by Ley; they were treated in the same manner as other convulsive diseases, and the whole died. The purport of Dr. Griffin's remarks is to show the insufficiency of Dr. Ley's theory for the explanation of the phenomena of the disease—*viz.* the pressure of enlarged cervical glands palsying the recurrents; but, as this opinion has now, we believe, few advocates, we need not detail the objections offered to it by the author.

" On the whole, after all the consideration I have devoted to the complaint, and having, I think, given other ingenious arguments of Dr. Ley their due weight, I must still confess myself a disciple of the older doctrine, that the affection is one of spasm or partial convulsion like cramp, rather than of paralysis. The fact of its being frequently benefited by antispasmodics, with which Dr. Underwood tells us he latterly cured most cases, and by anodynes, as opium, hemlock, cicuta, &c., recommended by all modern writers on the disease, favours this view; the circumstance of the sudden occurrence of the gasping and crowing on washing with cold water, laughing, crying, or agitation of mind, also supports it, as well as the almost universal co-existence of the carpo-pedal contractions, and the frequent termination of the complaint in convulsions. But above all these, as a strong analogical evidence for its spasmodic character, I place its paroxysmal nature and the manner in which the paroxysms occur. The office of the superior laryngeal nerves would lead us to expect a disposition to spasmodic action on the least irritation or excitement, recurring at irregular intervals, dependent of course on the return of the irritation or excitement, but far more on the increase or decrease of the susceptibility of the parts, and disposition to spasmodic action.

If it be inquired further, why such a dangerous result as the suspension of respiration in the crowing disease does not then occur more frequently, it can only be replied, that we are wholly ignorant of the morbid condition which disorders the functions of those nerves, or whether it exists at their extremities, or their origin in the medulla oblongata. If we suppose the affection to be organic, we should find it more difficult to account for the occasional recoveries under very mild treatment, than the usual fatality under the most active. If it be functional and therefore symptomatic, we can better understand why it might depend on a variety of causes; at one time upon an affection of the head, at another of the bowels, at another upon dentition; we can comprehend, too, how these several affections, influencing peculiar predispositions, may in one child occasion hydrocephalus, in another convulsions, in a fourth, that more rare infantile disorder, the crowing disease. This seems to be very much the view taken of the affection by Dr. Marsh, and more lately by Dr. Stokes. The disease, as the latter physician observes, may shew itself as a simple spasmodic affection of the larynx, *independent of any other perceptible lesion;* but this is the rarest case. In others, it is connected with the *irritation of dentition or deranged digestive functions;* while in a third class, it is symptomatic of *primary cerebral disease.* In this last the spasm of the glottis is as symptomatic of the cerebral disease as the convulsions of the extremities." P. 137.

In the *treatment* of this dangerous disease we must therefore search out for any disordered function, and apply the requisite measures, always taking care not to lower the tone and strength of the system injuriously. Indeed, the maintenance of the general health is of the first consequence, and to this end the management of diet and change of air are most important means. Dr. Griffin suggests that, in this and other diseases, especially such as are endemic, the placing the patient in an atmosphere as different as possible from that in which the disease originated, might often be beneficial. He recommends the child should be much in the open air, and sleep in a cool well-ventilated bed-room. He does not approve of the endeavour to treat the disease as a pure cerebral affection, and recommends tonics or antispasmodics and anodynes, according as debility or irritability may be the prominent feature. When there is no cerebral or abdominal disorder, iron or zinc may be employed in the endeavour for preventing a return of the attack, and the Indian hemp may be tried as an anodyne. The strumous character of these patients also indicates the use of iodine. Dr. Griffin truly observes that both the pathology and treatment of the affection are as yet quite unsettled; and thus sums up the amount of our present information respecting ascertained facts.

"By the concurrent testimony of almost all who have noticed the affection, it occurs for the most part, if not wholly, in strumous habits. It is frequently found in connexion with enlarged glands in the neck, and perhaps in the thorax. It is frequently found in connexion with eruptions on the face, ears, or scalp. It frequently terminates in convulsions, and is sometimes, though very rarely, ushered in by them. It is met with in families in which children are subject to head-affections or convulsions, but who have also the strumous disposition. It is sometimes met with in connexion with an apoplectic or comatose state from the commencement, as in the cases of crowing apoplexy which I have described. In a great proportion of the cases which terminated fatally there was no symptom of head-affection through their whole course, beyond the occasional fits of breathlessness and crowing: and the children were as well, apparently, a few moments before death, as they were previous to the first attack of the disease, or as any children could be. The complaint is sometimes, but rarely, attended by cough or permanent difficulty of respiration. I believe it may be said that, from one-third to half of all the cases of which we have any account, terminated in death. But this great mortality may perhaps, in some degree, be attributable to the over-active treatment pursued in many instances, from mistaken notions of the congestive or cerebral nature of the disease." P. 140.

Dr. Griffin makes no mention of the enlargement of the Thymus Gland, so frequently met with in this disease as to have obtained for it in Germany the appellation of "Thymic Asthma."*

---

* See Med. Chir. Rev. No. 57, p. 230, and Lancet, May 22, 1841.

7. *What are the Therapeutic Effects of Opium?*—Dr. Griffin employs this drug very largely, and speaks even enthusiastically of its virtues in various parts of his work. The present chapter contains some very useful observations upon its administration in different conditions of disease. It is universally allowed to be the most certain of the *narcotics*, but, in particular idiosyncracies, so far from composing the system, it exerts quite a contrary effect; and indeed we have felt somewhat surprised lately at the number of persons in whom we have observed this to be the case. In these cases, Dr. G. observes, although as a general rule we should abstain from giving opiates at all, it sometimes happens that some pressing symptom calls for their administration. He has found the vomiting and retching which occurs in some, prevented by a grain or two of capsicum, and the exciting effects observed in certain idiosyncracies are to be combated by combining the opiate with camphor, ipecac. or emetic tartar.

"When the results are sinking and faintness without stupor, they commonly arise many hours after the exhibition of the medicine, and are in fact mere symptoms of exhaustion, following the declining influence of an over-dose. They may be removed or alleviated by renewing the opiate in a smaller dose, or, if other considerations render this unadvisable, by stimulants frequently repeated, and eventually by sleep. It is often of great importance in medical practice, and especially so in all dangerous cases where opiates may have been largely administered, that we should remember a period of sinking and exhaustion is likely to come on from 12 to 24 hours after opiates have been discontinued. It almost invariably occurs, where the opiates have previously been long-continued, or taken in frequently-repeated doses, or where there is great constitutional susceptibility to their action."

Dr. Griffin alludes to cases in which great sinking and exhaustion followed the omission of even a very reduced dose. He cautions the physician, therefore, not to leave off opiates too abruptly, and not to attribute the debilitating effects which follow their too sudden withdrawal to the natural phenomena of the disease, and thus resort to improper means for relieving them.

"This is particularly apt to occur in the cases of young children or infants, when the violence of the inflammatory symptoms has gone by, and it has been found necessary to administer opiates. About the time the influence of the opiate is wearing out, if it has been a large one, the eyes look sunk, the eyelids lie half open, disclosing a small portion of the white cornea, the face is deadly pale, the skin clammy, and the whole appearance of the child suggests an apprehension that it is dying. Yet, if a little nourishment, or some slight stimulant, be given, or even if a little time be allowed to elapse, the heart will recover its tone, the little patient will revive, and may, perhaps, appear finally to be even in an improved condition."

This is a very faithful portrait of what we have repeatedly observed after the administration even of moderate doses of Dover's powder to young infants.

It is familiarly known that an *under-dose* of opium will disturb, while a full dose will compose; but Dr. Griffin draws attention to the important fact, that an *over-dose* will produce precisely the same ill-effects as an under-dose. Where a restless night has resulted from too much opium being given, sound sleep may occur the next night without any opiate whatever. So, too, if the doses of opium, given during the existence of inflammation with advantage, are continued when this has subsided, stupor or troubled sleep, according to the degree of surplus given, will result. "I am anxious to direct the attention of the profession to the consequence of an over-dose particularly, because I have seen physicians, under the circumstance, draw a directly opposite inference from it, and imagine they had given too small a quantity of the drug, when, nevertheless, on being induced to forego its repetition on the ensuing night, the patient has enjoyed a long and profound sleep."

An important question in the administration of opium as an *anodyne* is thus put by Dr. Griffin. *When a large dose has been administered, and a patient is still suffering intense pain, how long should one wait before it could be considered safe to repeat it?* He believes half an hour is the limit within which crude opium will be found to manifest its effects—the liquid preparations of the drug acting somewhat sooner: and that the dose may be then safely repeated if the pain or spasm is not relieved, however often it may have been given. This will not apply to *moderate* doses given to procure sleep, which first produce an exciting effect. Indeed, in susceptible constitutions the opiate should be given some hours before bed-time, in order that this excitation may have completely passed off. Another rule for the administration of opium for the relief of pain, especially of a periodical character, is, that a third part of the dose which was required to relieve the paroxysm is required to prevent its recurrence. "In fact, a moderate dose given in the interval, will sometimes prevent the accession of the fit, when no quantity, however great, can controul it, after it has once set in."

Of the *antiphlogistic* powers of opium, Dr. Griffin expresses his high opinion in many parts of this book: and states that for many years he has almost entirely relied upon it for the subdual of enteritis and peritonitis. He bleeds first, however, in subjects who will bear depletion, employing calomel also when the disease is very intense, and resists opium powerfully—suspending this as soon as the symptoms give way, and giving the opium alone, whereby troublesome salivation is usually prevented. In *rheumatic* inflammation it is as useful. In acute inflammation of the *mucous membranes* opium was formerly supposed to be contra-indicated; but the cases published by Dr. Stokes and others of cure of inflammation of the mucous membrane of the bowels with exhausting diarrhœa, shew the propriety of the practice. Dr. Griffin attributes the evil effects which have followed the use of opium in these inflammatory affections to its having been given in too small doses, which, in cases of mucous phlegmasiæ, suppress the diarrhœa without subduing the inflammation to which this had even acted as a relief. But in these, as well as in serous inflammations, bleeding must be premised where the strength will admit of it, applying at the same time warm poultices to the abdomen. In other cases, in which bleeding is out of the question, and opium seems powerless, as in bad dysentery, a combination of ipecacuan and opium acts sometimes surprisingly. Dr. Griffin alludes to two cases, in which he gave three grains of opium with from three to five of ipecacuan every two hours with the best effect.

Opium, producing in healthy persons a congestion of the brain, is contra-indicated in inflammatory affections of that organ and of the spinal-marrow; but when it is "combined with tart. emet. in sufficient doses it seems to possess an extraordinary power of allaying nervous irritation, quieting increased action in the capillary vessels, and inducing sound and refreshing sleep. In very many cases of puerperal mania, of delirium tremens, and in the delirium which arises in the advanced stages of fever, its effects are wonderful. Medical science is much indebted to Dr. Graves for having directed the attention of the profession to this most valuable combination, and pointed out so accurately its proper application."*

Dr. Griffin believes the great restorative power which opium possesses in *exhaustion from hæmorrhage* depends principally upon its property of producing congestion in the brain, and thus restoring tension to the cerebral vessels. In imminent danger five grains should be at once given, and two or three every hour or half-hour "until the pulse becomes distinct, the breathing quieter, and the tossing or flinging about in bed is allayed." Internal and external stimuli are of course to be conjoined with it.

We have extracted largely from this valuable chapter; for, although perhaps the author may somewhat over-rate the powers of this remedy, we feel convinced, from our own observation, that these are not sufficiently appreciated by the profession at large; and that the more the properties of this invaluable drug are inquired into the greater *will be the estimation in which it is held.* The rules and cautions given by Dr. Griffin are very important; for many of the evil effects supposed to have arisen from the inappropriateness of the remedy, have done so, in reality, from the timidity or want of discrimination of the prescriber.

8. *What Principles should regulate the Treatment of Hæmoptysis?*—The object of this paper is not to enter into an examination of the general subject of the treatment of this disease; but to protest against the excessive use of depletion and low diet in severe and repeatedly-recurring hæmorrhage. Dr. Griffin observes that he was first led to contemplate the unsatisfactory nature of this practice by comparing the cases in which it was resorted to, with others that presented themselves at the Dispensary, in many of which, immense quantities of blood had been coughed up many months before, the patients being still alive, not having observed, or having refused compliance with, the lowering treatment. Alluding to a case, in which all nourishment was refused to a patient dying of hæmoptysis to the last, he says—

"Of one thing I am convinced, that in all such diseases the patient should be allowed to die of the hæmorrhage, rather than of the debility following the interdiction of all food. Neglected cases daily prove to us, that even very alarming hæmorrhages may not prove immediately fatal although no abstinence be practised, while no one can doubt the certainty of speedy death if blood is daily bursting from the lungs, while all possible supplies in the way of food are cut off from the circulation. Setting aside however, altogether, those extreme cases

* "Dr. Graves directs 4 gr. of Emet. T. and 2 drachms of Laudanum to be mixed with half-a-pint of camphor mixture; two tablespoonfuls of which are to be given for the first dose, and one every half-hour afterwards, until the delirium abates, or some signs of drowsiness appear."

in which no possible treatment can succeed, I am still strongly impressed with the belief, that the principles upon which this rigid abstinence is imposed, is in almost every instance of protracted hæmorrhage an erroneous one." P. 206.

The hæmorrhage, active at first, becomes, sooner or later, passive, when its suppression is to be sought from means capable of giving tone to the capillary vessels; and thus menorrhagia and other uterine hæmorrhages, allayed by depletion at first, eventually often require stimuli and chalybeates. It is true, after the heart and vessels have recovered their tone, hæmorrhage may again be excited; but this has reference rather to the extent to which nutriment is given than to the principle upon which it is allowed or withheld. Those practitioners who are deterred in consequence of observing occasionally a recurrence of hæmorrhage after giving food, should observe—

"That, to form a fair inference on the subject, it should be considered, that at the commencement of such cases, recurrences of hæmorrhage often take place, perhaps every third or fourth day, whatever treatment is adopted; that the excitement occasioned by the nutriment is often rather the effect of a morbid irritability of the vascular system, arising from prolonged starvation than of its direct stimulant power; that this irritability, like the hæmorrhagic action of the pulse following extreme losses of blood, will diminish in proportion as we can gradually restore strength to the system by the administration of the mildest nutriment; and that it should more properly suggest the necessity of giving sedatives to allay it, than of recurring to a system of prolonged starvation, which in the highest health no constitution could resist, and under which in disease no reparative process could possibly be accomplished." P. 208.

Dr. Griffin adverts to the danger there exists in diminishing the tone of the capillaries, of congesting the bronchi or lungs, or inducing infiltration of blood. Moreover, this excessive depletion has a direct tendency to induce pulmonary consumption, by deteriorating the vital powers.

"There is the less defence for the practice I am contending against, as we are not without remedial agents which are little inferior to bleeding for the arrest of hæmorrhage, yet leave no permanent debility. I have seen very frightful cases of hæmoptysis subdued by an emetic of Ipecac., and by the continued administration of it afterwards in grain or two-grain doses every hour. Emetic tartar has also been successfully employed, and, when once the attack has assumed a passive character, it is well known that many other remedies prove highly influential." P. 210.

9. *How should Acute Rheumatism be treated.*—In passing the various modes of treatment under review, Dr. Griffin observes that *free depletion,* from a mistaken supposition of the identity of the disease with ordinary inflammation, is discountenanced more and more as our experience of the disease increases. "It will indeed appear that the average period occupied in the cure by it is longer, the success more doubtful, the pain more considerable, the danger of metastasis and of relapse greater, and the convalescence more tedious, on comparing it with other methods to which we shall presently advert." *Colchicum,* aided by moderate depletion and antiphlogistic regimen, is generally successful. Dr. Griffin has occasionally found, as well as Dr. Hope, obstinate and even dangerous diarrhœa attend its use, and by no means considers it either the most certain or most safe remedy. *Tartar-emetic* has been given by Dr. Griffin in even larger doses than Laennec and Louis employed, viz. a grain every hour for many consecutive hours. The first dose or two were usually vomited, but if tolerance then ensued the cure was often astonishingly quick, in cases even of many days' prior duration. Diarrhœa sometimes occurred, but it was generally easily arrested. It failed in its effects, however, in more than two-thirds of the cases. "On the whole it appears evident, that while emetic tartar administered in large doses occasionally displays a singular and extraordinary controul over acute rheumatism, we are yet at a loss to determine in what cases it is more particularly successful, or how its influence can be more extensively applied."

The author next adverts to the modes of treatment employed by Drs. Corrigan and Hope as far superior, as regards their certainty, the time occupied, and the freedom from metastasis or relapse, to any others. In all these advantages the one is little superior to the other; but in some cases the one, and in some cases the other plan may be most advantageously adopted, while in a third category the two modes may have to replace each other. Dr. Corrigan's treatment consists in the administration of one or two grains of opium every second or third hour, or of ten, twelve or more grains in the twenty-four hours. He dwells upon the importance of the dose being thus sufficient and full, a less quantity, given seldomer, only producing the stimulant and injurious effect of the drug.

"The opium should always be increased in dose, both as to frequency and quantity, until the patient feels decided relief; and should be then kept up at that dose until the complaint is steadily declining. The first indication that tells the practitioner that he has reached the proper dose, is the statement of the patient, who, in reply to an inquiry as to how he has passed the night, probably says that he has not slept, but that he is free from pain, and feels comfortable. This effect having been attained, the opium may then be continued in repetitions of the same dose as to frequency and quantity."—*Corrigan.*

Dr. Griffin has, however, found in some cases stupor, headache, and constipation (although in many others the bowels continued quite regular), obliging him to have recourse to Dr. Hope's plan. This consists in the administration (after one or two bleedings in the robust only), of gr. v ad x hyd. sub. with $1\frac{1}{2}$ ad 2 gr. opium every night, and a purgative, which will act four or five times at least, every morning. The following draught, given three times a day, expedites the cure. ℞. Vin. Colch. ♏x-xx. Pulv. Ipec. co. gr. v. M. Salin ℥x. Syr. ʒj. M. When the pain and swelling are much abated, which usually happens within two, and almost always within four days, and before this, if any tenderness of the gums occurs, the calomel is omitted, continuing one grain of opium at bed-time, and in some cases at noon, as also the colchicum draught, and morning senna purge. Dr. Griffin observes that, where a sore-mouth supervenes, the cure will have to be completed by the opium plan; or, if there is not much pain left, by quinine and potass. hydr. "Salivation can never occur in these cases, any more than in abdominal inflammation, or in cholera, unless the use of calomel be persevered in after the symptoms have completely given way, and a cure in part effected. So long as acute disease lasts, mercury will not salivate, but a single dose given after the disease has given way may do so, and the difficulty of avoiding it generally arises from the influence of our own apprehensions, which tempts us to continue the remedy beyond the absolute necessity, for fear of a relapse."

Disease of the heart is rare under either of these modes of treatment; but when discovered, large and repeated doses of calomel and opium are essential. When the rheumatism threatens a chronic form, or the attendant fever assumes a hectic character, quinine or the hydriodate of potass in full doses are of great use.

There are yet three other papers in the volume, whose analysis want of space forbids our undertaking. One of these, from the pen of Dr. D. Griffin, consists in a refutation of the usually received law of visible direction. The second is a long and interesting essay upon the questions, "Does suffering necessarily imply self-consciousness? Are sentient beings necessarily percipient?" in which Dr. Griffin opposes the deductions of those physiologists who assume the identity of consciousness and sensation, and hence the absence of the latter in the decapitated animal, and endeavours to explain the various movements, &c. in this condition as merely automatous or instinctive, however strong the expression of animal suffering may seem. He likewise criticizes the somewhat hasty generalizations of Dr. M. Hall. The last paper, entitled "Observations upon the application of Mathematics to the Science of Medicine," is a laboured recommendation of the Numerical Method, without, however, as far as we can perceive, advancing any new argument in its favour, or meeting successfully the objections which have been made to its adoption as a guide to practice.

Dr. Griffin is correct in stating that English practitioners have acquired a great distrust of the Method from one of the first fruits of its adoption by M. Louis, viz., the astounding conclusion that "bleeding is injurious rather than beneficial in inflammation of the lungs!" He explains the source of the mistake of this celebrated pathologist; but we must not lose sight of the important cautionary lesson; for if so grave an error can occur during the working of the system in the hands of its inventor, aided by the co-operation of a numerous corps of zealous and trained disciples, acting too in so favorable a field for observation as a large hospital—what must we expect from the endeavour to carry its application into the investigation of more complex diseases, by the hands of practitioners possessed of every variety of competence, acting independently of each other, and hardly possessed of forms of expression in common. Of all dangers a fallacious certainty is the greatest.

The lengthened notice we have bestowed upon Dr. Griffin's work proves the value we attach to its contents; and certainly any success it may meet with will be due to its own intrinsic merits and not to any care his publisher has bestowed upon it. We, however, say nothing about its general getting up; for we think that unnecessary expence is often incurred in sending out medical books in a style more fitting for the drawing-room library than for the practitioner's bookshelf; but we must protest against the disgraceful printing of the work, the innumerable typographical errors which crowd every page, rendering its perusal actually painful to the eye.

